# A Bivariate Twin Study of Lifetime cannabis Initiation and Lifetime Regular Tobacco Smoking Across Three Different Countries

**DOI:** 10.1007/s10519-024-10190-1

**Published:** 2024-07-30

**Authors:** Stephanie Zellers, Jenny van Dongen, Hermine H.M. Maes, Miina Ollikainen, Fang Fang, Scott Vrieze, Jaakko Kaprio, Dorret I. Boomsma

**Affiliations:** 1 Institute for Molecular Medicine Finland, University of Helsinki, P.O. Box 20, Helsinki, 00014 Finland; 2 Department of Biological Psychology, Vrije Universiteit, Amsterdam, The Netherlands; 3 Virginia Institute for Psychiatric and Behavioral Genetics, Department of Human and Molecular Genetics, Psychiatry and Massey Cancer Center, Virginia Commonwealth University, Richmond, VA USA; 4 Minerva Foundation Institute for Medical Research, Helsinki, Finland; 5 GenOmics and Translational Research Center, Research Triangle Institute International, Research Triangle Park, NC USA; 6 Department of Psychology, University of Minnesota, Minneapolis, MN USA

**Keywords:** Genetic correlation, Substance use, Twin model, Liability threshold model

## Abstract

Regular cigarette smoking and cannabis consumption are strongly positively related to each other, yet few studies explore their underlying variation and covariation. We evaluated the genetic and environmental decomposition of variance and covariance of these two traits in twin data from three countries with different social norms and legislation. Data from the Netherlands Twin Register, FinnTwin12/16, and the Minnesota Center for Twin Family Research (total *N* = 21,617) were analyzed in bivariate threshold models of lifetime regular smoking initiation (RSI) and lifetime cannabis initiation (CI). We ran unstratified models and models stratified by sex and country. Prevalence of RSI was lowest in the Netherlands and prevalence of CI was highest in Minnesota. In the unstratified model, genetic (A) and common environmental factors (C) contributed substantially to the liabilities of RSI (A = 0.47, C = 0.34) and CI (A = 0.28, C = 0.51). The two liabilities were significantly phenotypically (rP = 0.56), genetically (rA = 0.74), and environmentally correlated in the unstratified model (rC = 0.47and rE = 0.48, representing correlations between common and unique environmental factors). The magnitude of phenotypic correlation between liabilities varied by country but not sex (Minnesota rP ~ 0.70, Netherlands rP ~ 0.59, Finland rP ~ 0.45). Comparisons of decomposed correlations could not be reliably tested in the stratified models. The prevalence and association of RSI and CI vary by sex and country. These two behaviors are correlated because there is genetic and environmental overlap between their underlying latent liabilities. There is heterogeneity in the genetic architecture of these traits across country.

## Introduction

Smoking cigarettes and cannabis use tend to co-occur. While these are two different substances with distinct effects, their relation is complex and multifaceted (Agrawal et al. 2012). Their co-occurrence can be due to multiple mechanisms, including shared genetic and environmental risk factors (common liability hypothesis; Hatoum et al. 2023; Iacono et al. 2008; Iob et al. 2021; Korhonen et al. 2012; Krueger et al. 2002; Lynskey et al. 1998; Palmer et al. 2013; van Leeuwen et al. 2011; Vanyukov et al. 2012; Young et al. 2000), as well as gateway mechanisms, where using one drug, such as cigarettes, may increase the likelihood of trying or using another drug, such as cannabis (Becker et al. 2015; Weinberger et al. 2020), or self-medication to ease symptoms of other disorders (Sumbe et al. 2022).

Studies of the genetic variability underlying tobacco and cannabis use and their association began primarily with twin studies. Twin studies are frequently conducted on each trait separately (Maes et al. 2004; Kendler et al. 2013; Smolkina et al. 2017; Hines et al. 2018), with some studies investigating their overlap (Maes et al. 1999; McGue et al. 2000; Kendler et al. 2003, 2005, 2008; Rhee et al. 2003; Fowler et al. 2007; Zellers et al. 2022). These studies investigating their overlap tend to focus on measures of substance involvement (frequency, heaviness of use, dependence, etc.) rather than initiation. The twin studies of initiation of both substances that we identified focused primarily on adolescence (Huizink et al. 2010; Agrawal et al. 2010, 2016) which is a critical period for substance use development (McGue et al. 2014). Each of these three studies found substantial positive genetic and environmental overlap in the liability to initiate tobacco and cannabis use.

Similarly, more recent molecular projects in genomics and epigenetics were sometimes carried out for each trait separately (Joehanes et al. 2016; Li et al. 2020; Xu et al. 2020; Hillmer et al. 2021; Pasman et al. 2022). Studies evaluating their overlap based on genome-wide genetic data exist and a common finding is that the genetic variants influencing the two traits are found to cluster in the same region of the genome or that the traits are genetically correlated (Agrawal et al. 2015; Stringer et al. 2016; Pasman et al. 2018; Allegrini et al. 2019; Chang et al. 2020; Johnson et al. 2020; Schaefer et al. 2023; Nannini et al. 2023; Fang et al. 2023). Both the twin and molecular studies investigating the association between tobacco and cannabis use indicate strong genetic overlap, but that genetic factors do not entirely explain the association, suggesting the importance of the environment.

Different social and cultural factors may also play a role in the use of tobacco and cannabis; for example, in the Netherlands cannabis has been available for recreational use in “coffee shops” since 1976 and is relatively destigmatized, but in Finland the sale and use of cannabis is entirely illegal. Cannabis policies vary across the United States, but policies have become increasingly permissive across the last two decades, with recreational sales permitted in about half of states. Indeed, there is evidence to show that in the USA the perceived harmfulness of cannabis use has decreased over time, social acceptability has increased, and that cannabis use has similarly increased in the same time period that these legal changes have occurred (Cerdá et al. 2012; Hasin 2018; Coughenour et al. 2021; Zellers et al. 2023). On the other hand, tobacco policies have become stricter globally across the last 40 years, but still vary between countries to some degree (Reubi and Berridge 2016). Just as the prevalence of cannabis use has increased with more permissive policies, the prevalence of tobacco use has decreased as policies become stricter (Helakorpi et al. 2007; Boardman et al. 2010; Flor et al. 2021). These results indicate that social and cultural factors influencing substance use are context dependent, and these factors differ not only across time, but also across geographic location. Therefore, studies of the cigarette and cannabis use and the relationship between them should be sensitive to cultural context.

Here we focused on lifetime initiation of regular cigarette smoking (RSI) and initiation of cannabis use (CI), in data collected from adults in large population based twin registers of European ancestry from three sites: the USA (Minnesota), the Netherlands and Finland. We evaluated the variance and covariance decomposition of lifetime smoking and lifetime cannabis use across cultural contexts. If we find differences across cultures, this could have important ramifications for the way we carry out multi-site studies of these traits.

The classical twin design is a powerful tool to address the etiology of individual differences and its bivariate extension allows to examine whether the co-occurrence of two traits is due to genetic or environmental shared risk factors. In a series of bivariate threshold twin models, we sought to address several research questions: (1) What degree of overlap is there between the underlying liabilities to RSI and CI? (2) What is the genetic correlation? (3) What is the environmental correlation and are the traits correlated due to the shared environment, unique environment, or both? (4) Do these values differ between the Netherlands, Finland, and USA? (5) Do these values differ between males and females?

We expected both traits to be heritable and we expected positive phenotypic, genetic, and environmental correlations between them, in all three countries. We also expected to replicate established sex differences, where males initiate at higher rates than females. Lastly, we anticipated some parameter differences in variance and thresholds for initiation between sexes and countries given the varied legal and social environments around cannabis and tobacco use, but we did not have directional hypotheses regarding country differences. The pre-registration is available at https://osf.io/utr6w/.

## Methods

### Participants

We harmonized data from twin cohorts across three countries (total *N* = 21,617): the Netherlands Twin Register (*N* = 12,987), FinnTwin12 and FinnTwin16 (combined *N* = 5,888), and the Minnesota Center for Twin Family Research (*N* = 2,742). All are longitudinal twin studies that have been described extensively elsewhere (Geels et al. 2013; Treur et al. 2017; Wilson et al. 2019; Rose et al. 2019; Kaidesoja et al. 2019; Ligthart et al. 2019). The Netherlands Twin Register, FinnTwin12 and FinnTwin16 assessed same-sex and opposite-sex twin pairs, whereas the Minnesota Center for Twin Family Research only recruited same-sex twin pairs. Sex is specifically sex assigned at birth and as reported on birth certificates. Furthermore, the Minnesota Center for Twin Family Research sample is composed of three cohorts with varying birth years, assessment structures, and assessment years.

We utilized one assessment per individual; assessments were selected to represent the most recent assessments and target a mean age of assessment in the twins’ 30s. Importantly, this mean age of assessment is beyond the normative ages of tobacco and cannabis initiation, which generally occurs between ages 15–19 (Richmond-Rakerd et al. 2016; Blanco et al. 2018). We utilized the most recent non-missing assessment from waves 8 and 10 in the adult Netherlands Twin Register (2009–2013), the FinnTwin12 wave 4 assessment (2006–2009), the FinnTwin16 wave 5 assessment (2010–2012), and the most recent non-missing assessment from waves 4, 5, 6, and 7 in the Minnesota Center for Twin Family Research (2013–2022).

### Measures

**Fig. 1:**
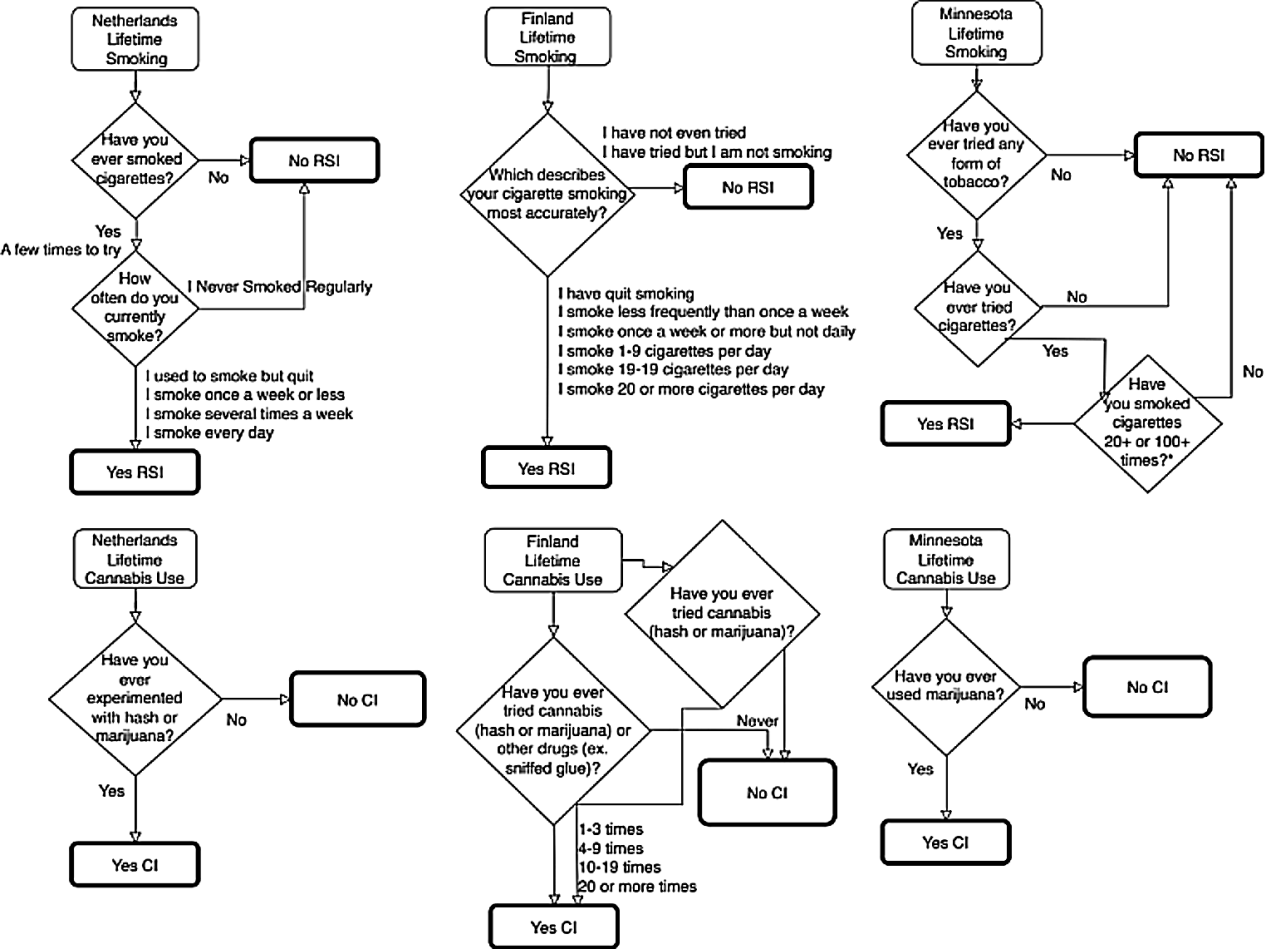
Flowchart of the binary phenotype harmonization from original questionnaire items and response options in each sample. Note that the items for Finland differed slightly between the FinnTwin12 and FinnTwin16 cohorts. RSI refers to regular smoking initiation, CI refers to cannabis initiation

Both RSI and CI were defined as binary measures indicating whether the individual had ever been a regular cigarette smoker (yes or no) and whether the individual had ever used cannabis (yes or no). Measures were harmonized between samples, as item wording and skip-outs varied. Figure 1 provides a flowchart of the questionnaire items, response options, skip-outs, and binarization for each sample. Importantly, the FinnTwin12 and FinnTwin16 items differed slightly, as one asked only about cannabis and the other asked simultaneously about cannabis and other drugs. For participants answering yes to “have you used cannabis or other drugs” we assume they have used cannabis and code as yes for CI. Sex was operationalized as a binary variable based on sex assigned at birth as reported at intake (male or female).

### Analyses

All analyses were conducted in RStudio. We used the R packages ggplot2 (Wickham 2016) for visualizations, OpenMx (Boker et al. 2011; Neale et al. 2016) for twin model variance decompositions, and Psych (Revelle 2023) for tetrachoric correlations. All data (i.e. from complete and from incomplete twin pairs in which only one twin replied) were analyzed.

We first obtained descriptive statistics including prevalence of endorsement for each binary trait by country and sex. Resemblance between twins and between traits were estimated by tetrachoric correlations. Prevalences were estimated separately for each country × sex group; tetrachoric twin correlations were estimated separately for each country × sex group and stratified by zygosity (monozygotic, same-sex dizygotic, and opposite-sex dizygotic). Given the differences in item wording for the Finnish sample and cannabis use, we compared the endorsement between the two items, to ensure that the inclusion of “cannabis or other drugs” was not upwardly biasing endorsement.

We then fitted a bivariate binary threshold twin model that included additive genetic (A), shared environmental (C), and unique environmental (E) variance components. Binary threshold models assume that the observed variables (ex. having initiated cannabis use or not) are indicative of underlying, normally distributed continuous liability. This latent liability has a threshold (the parameter that depends on the prevalence) above which the trait is observed, and below which it is not. First, all data were analyzed simultaneously in one model; in other words, the model was not stratified by sex or country and produced one set of parameter estimates. We identified the binary threshold models by fixing the mean of the liability distribution to zero and the variance to one and estimated the threshold, which can be interpreted as a z-score. Additionally, we included a covariate of age on the mean of the liability distribution and computed predicted thresholds at a set of ages covering the middle of the countries’ age distribution. We retained all ACE parameters to avoid bias in the point estimates.

Next, as our research questions include the investigation of country and site differences, we ran the same bivariate binary model stratified by country and sex. This stratified model produced six sets of parameter estimates (ex. Unique parameter estimates for each country -sex group: Netherlands males, Netherlands females, Finland males, Finland females, Minnesota males, and Minnesota females). We then compared parameter estimates between these six sets to evaluate country and sex differences. The main parameter of interest was the genetic correlation between the two liability dimensions, but all possible parameter differences were investigated.

Model comparisons were conducted via likelihood ratio test, in which parameters of interest were fixed to equality in a reduced model, and the fit of this reduced model was compared to the full model where the parameter was free to vary between groups. If the equality constraint resulted in a significant worsening of model fit, the parameter was interpreted as being different between groups. If the equality constraint did not significantly worsen model fit, as indicated by the likelihood ratio test, then that parameter was interpreted as not significantly differing between groups.

Therefore, to evaluate country differences, we compared the full model to a model in which the parameter of interest was fixed to equality between males across the three countries and between females across the three countries (ex. Comparing Netherlands males to Finland males to Minnesota males in a 2-df test). To evaluate sex differences, we compared the full model to a model in which the parameter of interest was fixed to equality between males and females separately for each country (ex. Comparing Netherlands males to Netherlands females in a 1-df test).

## Results

Descriptive statistics for age at data collection, year of data collection, sample sizes, sex, zygosity, and trait endorsement for each country are presented in Table 1.

**Table 1:** Demographics and descriptive statistics by sample

	Netherlands	Finland	Minnesota
N. Individuals	12,987	5,888	2,742
Age range	17–97	22–37	22–49
Age mean (SD)	32.2 (14.8)	31.3 (4.2)	36.2 (6.9)
% Female	67.2%	56.9%	55.2%
% MZ	47.0%	32.2%	62.2%
% OS DZ	25.3%	35.5%	0%
Years of data collection	2009–2013	2006–2012	2013–2022
% Yes RSI	33.2%	47.5%	44.7%
% Yes CI	29.5%	25.7%	62.9%

*Note* *N* refers to number; *MZ* refers to monozygotic; *DZ OS* refers to opposite-sex pairs dizygotic pairs; *RSI* refers to regular smoking initiation; *CI* refers to cannabis initiation

**Fig. 2:**
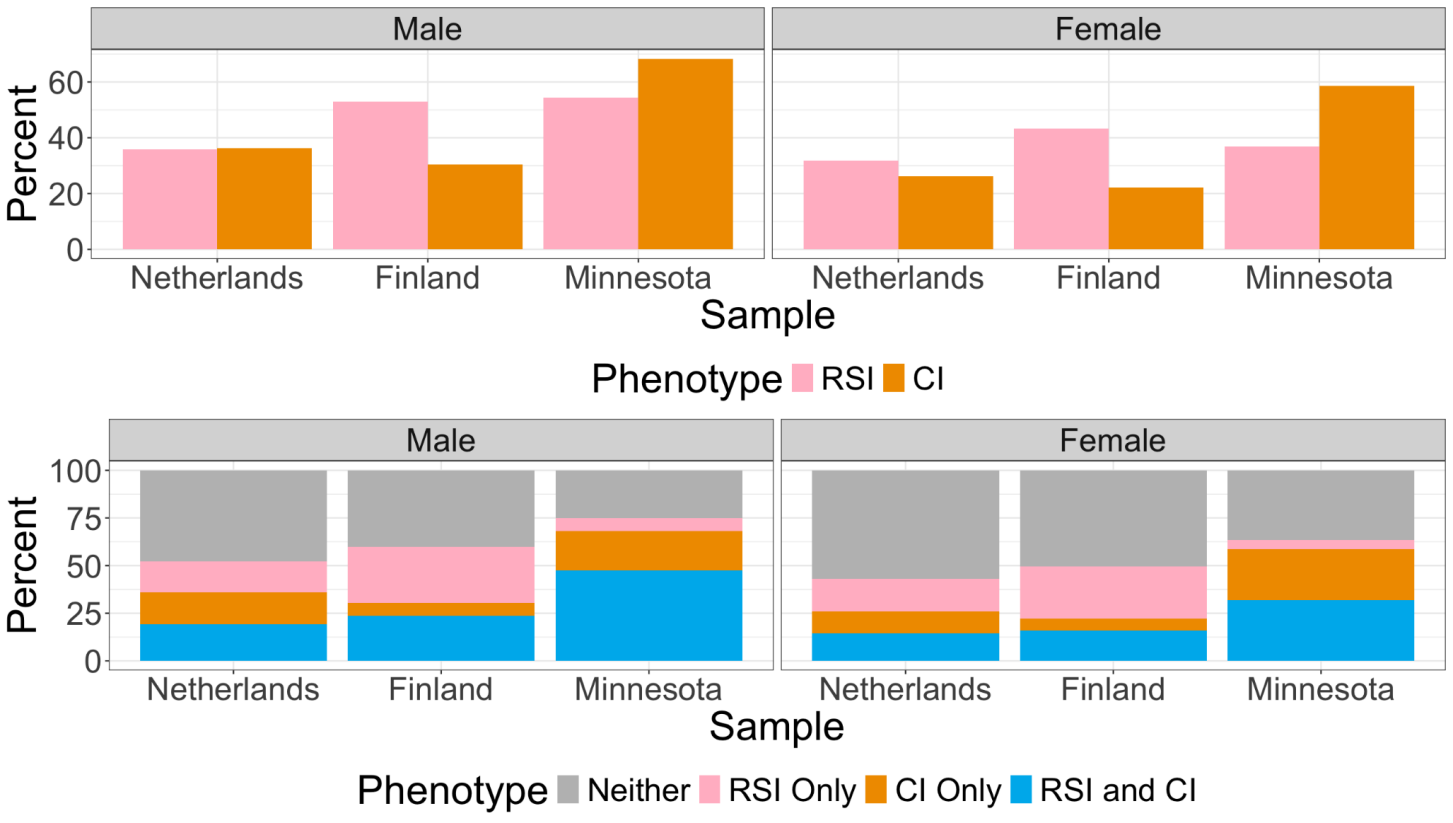
Bar charts depicting the prevalence of lifetime smoking and lifetime cannabis use stratified by sex and sample. The top bar chart presents the prevalence of each phenotype separately. The bottom bar chart presents the percentages of individuals who have endorsed neither phenotype, endorsed both phenotypes, and endorsed one phenotype but not the other. *RSI* refers to regular smoking initiation, *CI* refers to cannabis initiation

All three twin cohorts have overlapping age ranges, with the age range being widest in the Netherlands and narrowest in Finland. Mean ages are comparable and in all three twin cohorts, the mean age is beyond the typical age of substance initiation. Further details on the prevalence of each trait are presented in Fig. 2.

Tetrachoric correlations are presented in Table 2. In most cases and as expected, traits in monozygotic twin pairs are more strongly correlated than in same-sex dizygotic twin pairs, and same-sex dizygotic twin pairs are correlated more strongly than opposite-sex dizygotic pairs. There are some groups for which the monozygotic and dizygotic twin correlations are comparable (RSI in Finnish males, CI in Minnesota females). For the two Finnish cohorts, prevalence of CI was not higher in the cohort asked about “cannabis use or other drugs” (24.8% responded yes) as compared to those asked only about cannabis use (27.7% responded yes), suggesting that the item wording was unlikely to be biasing endorsement.

**Table 2:** Tetrachoric twin correlations by sex and sample

		MZ M	DZ M	MZ F	DZ F	DZ OS
RSI	Netherlands	0.86	0.52	0.82	0.55	0.49
	Finland	0.78	0.71	0.85	0.67	0.40
	Minnesota	0.76	0.51	0.77	0.54	NA
CI	Netherlands	0.73	0.64	0.73	0.49	0.51
	Finland	0.74	0.47	0.83	0.61	0.33
	Minnesota	0.84	0.60	0.69	0.73	NA

*Note* *MZ* refers to monozygotic pairs; *DZ* refers to dizygotic pairs; *M* refers to same-sex male pairs; *F* refers to same-sex female pairs; *OS* refers to opposite-sex dizygotic pairs; *RSI* refers to regular smoking initiation; *CI* refers to cannabis initiation

Based on the tetrachoric correlations, we expected to see additive genetic influences on both traits. We evaluated this in the unstratified model, where we pooled across country and sex (13 estimated parameters, df = 31,325, AIC = 35026.61). As expected, RSI and CI are both heritable (RSI A = 0.47 [95% confidence interval = 0.36, 0.58], CI A = 0.28 [0.22, 0.39]) and had a substantial shared environmental component (RSI C = 0.34 [0.24, 0.44], CI C = 0.51 [0.39, 0.60]). The two traits were positively correlated at the phenotypic level (rP = 0.56 [0.54, 0.58]) and the decomposed correlations were also all moderate to strong (rA = 0.74 [0.54, 0.93], rC = 0.47 [0.45, 0.63], rE = 0.48 [0.39, 0.58]) indicating both genetic and environmental overlap in the propensity to RSI and CI. We followed this up with the stratified model to evaluate country and sex differences.

**Table 3:** Bivariate twin model results split by sex and sample

		Netherlands		Finland		Minnesota	
		Male	Female	Male	Female	Male	Female
RSI	A	0.83 [0.01, 0.85]	0.66 [0.42, 0.82]	0.07 [-0.14, 0.84]	0.68 [0.01, 0.77]	0.60 [-0.70, 1.00]	0.45 [0.13, 0.54]
	C	-0.01 [-0.02, 0.69]	0.15 [0.01, 0.39]	0.68 [-0.01, 0.77]	0.18 [-0.11, 0.70]	0.17 [-0.25, 0.51]	0.31 [-0.01, 0.61]
	E	0.18 [0.12, 0.24]	0.19 [0.16, 0.24]	0.25 [0.17, 0.42]	0.14 [0.11, 0.22]	0.23 [0.16, 0.33]	0.23 [0.16, 0.33]
CI	A	0.26 [0.01, 0.81]	0.52 [0.01, 0.78]	0.70 [-0.08, 0.77]	0.41 [-0.01, 0.49]	0.54 [0.17, 0.63]	-0.06 [-0.37, 0.89]
	C	0.47 [0.11, 0.57]	0.20 [-0.10, 0.59]	0.04 [-0.19, 0.78]	0.40 [-0.02, 0.60]	0.29 [-1.00, 0.64]	0.75 [0.48, 0.83]
	E	0.28 [0.22, 0.34]	0.27 [0.22, 0.35]	0.26 [0.16, 0.38]	0.19 [0.10, 0.27]	0.16 [0.02, 0.26]	0.31 [0.24, 0.45]
Cor.	Phen.	0.58 [0.54, 0.62]	0.58 [0.55, 0.61]	0.51 [0.45, 0.56]	0.51 [0.46, 0.56]	0.70 [0.57, 0.76]	0.69 [0.64, 0.75]
	A	1.00 [0.89, 1.00]	0.55 [0.51, 0.61]	1.00 [-1.00, 1.00]	0.69 [-0.21, 1.00]	0.70 [-1.00, 0.75]	NA
	C	NA	0.85 [-1.00, 1.00]	1.00 [-1.00, 1.00]	0.31 [-1.00, 1.00]	1.00 [-1.00, 1.00]	0.81 [-1.00, 1.00]
	E	0.44 [0.22, 0.56]	0.48 [0.34, 0.69]	0.48 [-0.24, 0.70]	0.36 [0.04, 0.47]	0.41 [0.10, 0.98]	0.67 [0.42, 1.00]

*Note* *A* refers to additive genetic component; *C* refers to shared environmental component, and *E* refers to unique environmental component; *Cor*. refers to the correlation between substances; *Phen*. refers to the phenotypic correlation. NA indicates a correlation that cannot be estimated due to one or more negative point estimates, - indicates bound did not converge. The first number refers to the point estimate and the number in brackets is the 95% confidence interval around that point estimate. *RSI* refers to regular smoking initiation; *CI* refers to cannabis initiation

**Fig. 3:**
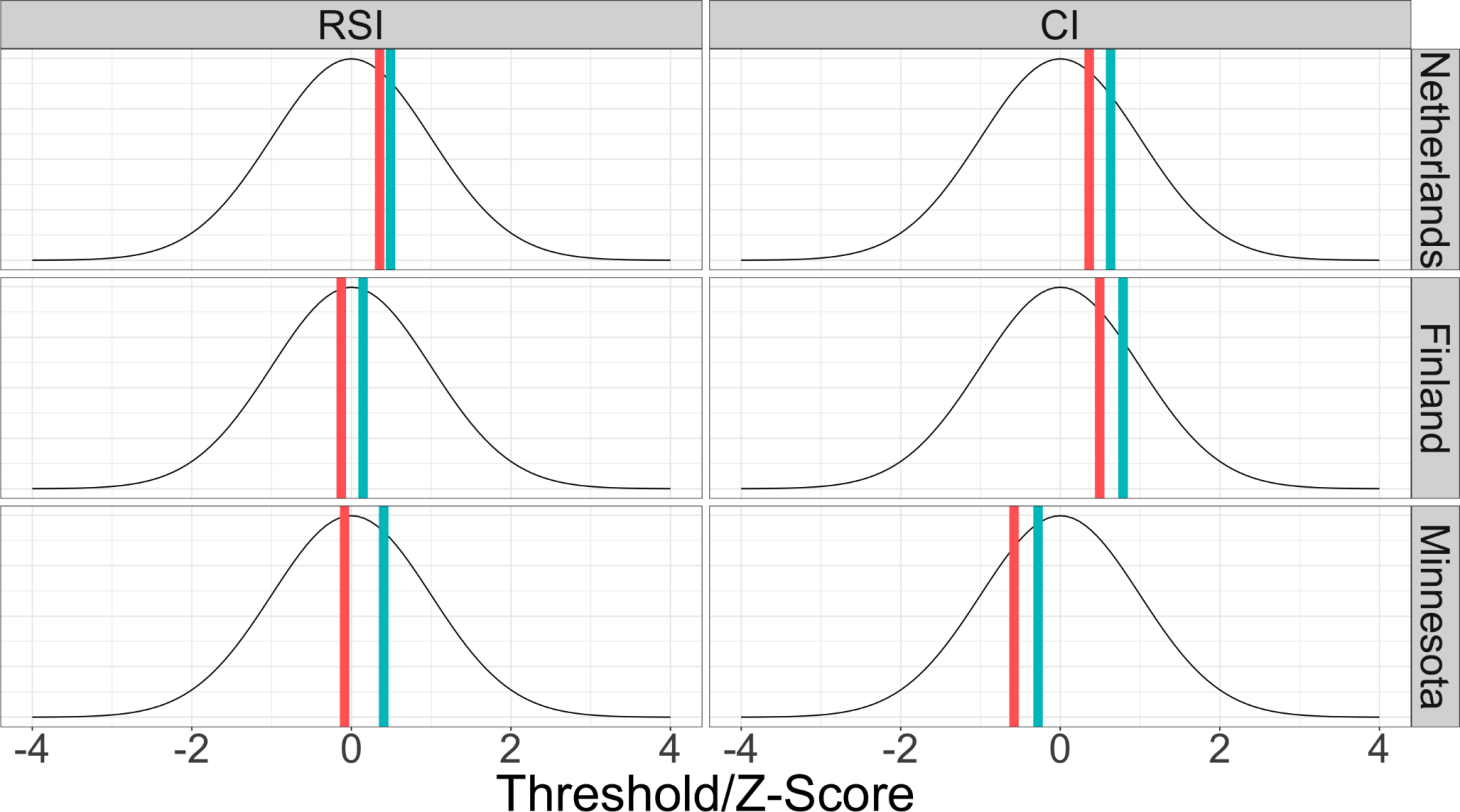
Diagram representing the thresholds for initiation of lifetime smoking and lifetime cannabis use, split by sample and sex. Blue lines represent the threshold for females and red represent the threshold for males. Thresholds can be interpreted as a z-score and are depicted here against a normal distribution with mean of 0 and variance of 1

The stratified model estimates and confidence intervals are presented in Table 3 and thresholds for initiation of each substance are presented in Fig. 3 (78 estimated parameters, df = 41,484, AIC = 45,554.98). The stratified (full) model fit better than the unstratified (reduced) model (χ^2^ = 10,398.37, df = 10,159, *p* = 0.047) As we observed monozygotic and dizygotic twin correlations of comparable magnitudes for RSI in Finnish males and CI in Minnesota females, we similarly found non-significant heritability for these traits. All other traits were heritable, though notably, confidence intervals for A and C estimates were very wide despite the large sample size. In turn, the decomposed correlations could not be stably estimated in the stratified models, with confidence intervals including +/- 1.

We identified country and sex differences in the thresholds for each trait. The thresholds for RSI and CI were significantly lower for males as compared to females, i.e. reflecting the higher prevalence in men. This was also true for all three countries (out of all six comparisons, the largest p-value was 1.2 × 10^− 5^). Thresholds also differed between countries, and this was true for both males and females and for both substances. With respect to RSI, the threshold was higher for Netherlands males (χ^2^ = 202.3, df = 2, *p* = 1.2 × 10^− 44^) as compared to both Finland and Minnesota males, which did not differ from each other (χ^2^ = 0.91, df = 1, *p* = 0.34). On the other hand, for females, the threshold for RSI was different between all three twin cohorts (χ^2^ = 131.9, df = 2, *p* = 2.3 × 10^− 29^); the threshold was highest in the Netherlands, lowest in Finland, and Minnesota was in between. With respect to CI, the threshold differed between males from all three samples (χ^2^ = 339.1, df = 2, *p* = 2.3 × 10^− 74^); the threshold was highest in Finland, lowest in Minnesota, and the Netherlands threshold was in between the other two. The same pattern was observed for females (χ^2^ = 435.5, df = 2, *p* = 2.6 × 10^− 95^).

Given the width of confidence intervals, we generally did not identify sex differences or country differences in variance decompositions for either RSI or CI. There were two exceptions where we identified sex and country differences, as evidenced earlier by the relative magnitudes of MZ and DZ twin correlations. RSI was less heritable in Finland males as compared to males from other samples (χ^2^ = 16.9, df = 4, *p* = 2.0 × 10^− 3^), and as compared to Finland females (χ^2^ = 12.4, df = 2, *p* = 2.0 × 10^− 3^). Similarly, CI was less heritable in Minnesota females, as compared to females from other samples (χ^2^ = 20.9, df = 4, *p* = 3.3 × 10^− 4^) and as compared to Minnesota males (χ^2^ = 6.9 df = 2, *p* = 0.03).

We also observed country differences in the more stably estimated phenotypic correlations. Phenotypic correlations did not differ between males and females within a country, but they did differ between countries for both males (χ^2^ = 21.4, df = 2, *p* = 2.2 × 10^− 5^) and females (χ^2^ = 22.6, df = 2, *p* = 1.3 × 10^− 5^). The phenotypic correlation between RSI and CI was strongest in Minnesota (rP ~ 0.70), weakest in Finland (rP ~ 0.45), and the estimate for the Netherlands was in between the two (rP ~ 0.59). Models to evaluate sex and sample differences in the decomposed correlations did not stably converge.

## Discussion

We estimated a bivariate twin decomposition of the variation underlying liability for RSI and CI. We investigated these decompositions in samples from three countries (the Netherlands, Finland, and Minnesota), which have differing policies around cigarettes and cannabis use. We identified important country and sex differences in the relationship between RSI and CI.

We replicated established sex differences in liability for RSI and CI; in all twin cohorts and for both substances, males were more likely to endorse RSI or CI as compared to females. These prevalences are captured in the twin model via the threshold for initiation, which can be interpreted as a z-score on the underlying latent risk dimension. If an individual’s latent risk is above the threshold, initiation is observed. Behaviors with higher prevalence in the population will have a lower threshold for initiation.

The thresholds for RSI and CI were higher for females as compared to males, aligning with the higher prevalence in males in all three countries. There were also country differences in the threshold for RSI for females but not males. With respect to CI, the thresholds also differed between all three countries for both males and females. The threshold for CI was lowest in Minnesota, highest in Finland, and in between for the Netherlands. These differences in threshold likely are driven by a variety of legal and cultural differences, including social norms around substance use, types of available products and administration methods, legal policies, and ease of acquiring of substances.

Legal differences are one potential source, as there are substantial differences in policies between the three countries. Cannabis use is illegal in Finland, is decriminalized for personal use in the Netherlands, and recreationally legal in about half of the US. Cannabis was not recreationally legalized in Minnesota, the birth state of all twins in the Minnesota sample, until 2023 but not all participants resided in Minnesota at the time of assessment. On the other hand, Finland and the Netherlands are considered on the forefront of tobacco control worldwide (Timberlake et al. 2020; World Health Organization 2023) as compared to the United States which lags behind (Action on Smoking and Health 2020). These important legal differences impact how easy it is to obtain cigarettes or cannabis, but also highlight the social acceptability of their use. Future work could also characterize how substance use relates to broader disinhibited behaviors in sites with different substance policies.

Trait correlations differed significantly by country but not by sex. RSI and CI were most strongly related in Minnesota and least strongly related in Finland. Previous developmental research has indicated that the strength of relationship between substances does vary by age (Vrieze et al. 2012; Zellers et al. 2022) and here we provide additional evidence that the strength of relationship may also vary by cultural context, though we cannot rule out age effects. Further, we cannot rule out differences by birth cohort, which could also reflect temporal changes in the use of substances over time. Smoking has become much less prevalent in the past decades, while cannabis use has increased, though trends also vary by country. Lastly, other studies have not identified cross-country differences in smoking initiation (Madden et al. 2004) and so the existence of cultural differences, or lack thereof, likely depends on a combination of age, period, and cohort effects.

This has important implications for broader research on substance use and related behaviors in the externalizing spectrum. Indeed, many studies have been published on the general genetic liability to substance use and externalizing behaviors, including some studies from Minnesota and Finland overlapping with the participants included in the present work (Young et al. 2000; Krueger et al. 2002; Dick et al. 2009; Agrawal et al. 2010; Edwards and Kendler 2012; Korhonen et al. 2012; Palmer et al. 2013; Karlsson Linnér et al. 2021). To our knowledge, only one study has harmonized and compared twin models of externalizing psychopathology and its relationship to cannabis use across samples; no sample differences were identified but the samples were American samples from different states (Zellers et al. 2020). As we have established that in adulthood, the covariation between substances differs between countries, future directions could therefore explore cultural differences in the relationships between substances and other externalizing behaviors. Lastly, we identified sex and country differences in the sources of variation and covariation underlying each trait, but these differences came with the large confidence intervals around the A and C variance estimates and should therefore be interpreted with care. Future work could attempt to identify specific cultural differences that may be driving the observed differences in prevalence and covariation between the two substances.

### Limitations

One measurement limitation is that the survey items are not the same across the twin cohorts and therefore we needed to harmonize variables. Some differences therefore could be artifacts of the harmonization process, particularly for RSI, as the items to measure this differed more substantially between countries as compared to the items for CI. Secondly, the definitions for initiation differed by substance; for RSI we considered initiation of regular use but for CI we considered initiation of any use. This could contribute to explaining differences in heritability between the two substances. Additionally, age and year of data collection varies between twin cohorts and also could contribute to identified differences.

An additional measurement limitation is that co-use of cannabis and tobacco (sometimes referred to as spliff use, blunts, or mulled cigarettes; Schauer et al. 2017) was not measured. Previous works have reported meaningful differences between individuals who co-use cannabis and tobacco, as compared to individuals who use only one substance, as well as individuals who do use both substances but not concurrently (Agrawal et al. 2012; Schauer et al. 2017; Tucker et al. 2019; Akbar et al. 2019; Kumar et al. 2020; Hindocha et al. 2021). Furthermore, there could be reporting bias introduced, as it is possible that individuals regularly co-use cannabis and tobacco would not be self-reporting as lifetime smokers, because the tobacco items specified cigarette use, rather than any tobacco use. That said, the vast majority of individuals who report use of tobacco-containing cannabis products also report other tobacco use, including cigarettes (Kumar et al. 2020), so any reporting bias introduced by our lack of co-use items is likely minimal.

Lastly, in twin models utilizing binary data, the variance components underlying twin similarity (additive genetic/A and shared environmental/C) are highly correlated and therefore can be difficult to tease apart. We used a direct symmetric model parameterization and elected to interpret the full ACE model results, rather than removing components that were not significantly different from zero, as to not upwardly bias the other components’ estimates (Verhulst et al. 2019). Because of this, our model resulted in comparatively wide confidence intervals around our A and C estimates in the stratified models, despite large sample sizes. Given these wide intervals, we were unable to meaningfully compare parameters of interest, such as the genetic correlations, across samples; all comparisons were nonsignificant despite some large differences in point estimates. Future work may evaluate traits with more variability, such as age at first use, age at regular use, frequency or quantity of tobacco and cannabis consumption, to avoid the limitations of working with coarser, binary data. Additionally, measures that more accurately gauge the heaviness of use or problematic use may be better indicators of underlying genetic liability to substance use and abuse, particularly under theoretical conceptualizations that treat substance use as one manifestation of broader externalizing liability.

### Conclusions

We identified meaningful sex and country differences in the liabilities RSI and CI. Our findings also have implications for the measures of substance use utilized in genetically informative studies. Whereas large genomic consortium studies benefit from coarse measures of use that can be readily harmonized across many participating studies, twin studies may benefit from including both binary and continuous measures of use. Furthermore, while the existence of general genetic vulnerability to substance (mis)use is supported across cultures and genetic ancestries (Saunders et al. 2022; Hatoum et al. 2023), the degree to which substances are phenotypically related may depend on cultural context.

## Data Availability

The research data used in this study are confidential and are not publicly available to protect participant privacy.
